# The Battle of the Clubs

**Published:** 1897-06

**Authors:** 


**The Battle of the Clubs.** By THE SPECIAL COMMISSIONER OF THE "LANCET." Pp. viii., 230. London: The Offices of the *Lancet*. [1895-96].

The thanks of the general practitioner throughout the land are due to the *Lancet* for such an impartial and comprehensive review of this—for a large number of doctors—the burning question of the hour. The facts connected with the societies which provide medical aid for the sick poor are taken from anywhere between Plymouth and York, the great hives of industry being naturally the most important, and the general position seems to be much about the same everywhere—only here and there being "more so." The picture disclosed is a gloomy one, though occasionally a ray of light and encouragement appears. Some few instances of medical success are recorded, and these show what might be done by loyalty, brotherhood, association, and above all by discipline.

It goes without saying that doctors are bad business people; they must, however, become good ones, and it is pretty well understood that now, whatever it may have been in former times, Heaven helps those only that help themselves; and the sooner it is recognised that self-reliance is their only effective weapon, the sooner will their deliverance come. Appeals to authorities, medical councils, and the rest are futile. There is no body of men in existence having the same power, the same opportunity of influencing public and private opinion, as doctors; and this influence must be exerted, not only to the utmost, but systematically. No battle is won without fighting: courage and self-denial are the essentials of success, and the example set at Cork must be kept in mind. During the battle the weaker brethren must be supported, money must be provided, leaders must be found and—a much more important matter—must be implicitly obeyed. The organisation must be on a national basis, and in fact the moral generally is the text so harped upon by the political agent to the constituencies, "Organise, organise, organise."

One or two special points may be noticed. Some are inclined to dogmatise as to the ease with which a wage limit standard might be fixed; but it would appear that thirty shillings a week by no means represents the same amount of wealth in all places. A reasonable discretion must be permitted. Doctors must compose their own committees of management, and the "honorary treasurer who is also chairman," and the "honorary member," must disappear. Some complain of the difficulty of bringing the "consulting" class into line. This is a difficulty which would certainly disappear if public opinion and personal interest pointed the same road. There would be some acrimony of course; but if this battle is to be fought, it must be "without gloves," or better not at all. As a basis and a starting point, the Bexhill Rules (p. 63) would be found an excellent model.

The malice and the skill and cunning with which the profession has been made to fight itself, as at Northampton, are a revelation. And at Birmingham it would almost seem as if some doctors had behaved as disgracefully as those who exploited them. It is impossible to close without paying a tribute of admiration to the unanimity and loyalty with which this question has been fought out at Brussels; the gallantry and self-denial with which the men stood shoulder to shoulder are beyond all praise. Shall we see anything like it in England? We doubt it.

